# Hypoxia and its possible relationship with endometrial receptivity in adenomyosis: a preliminary study

**DOI:** 10.1186/s12958-020-00692-y

**Published:** 2021-01-08

**Authors:** Song Guo, Di Zhang, Xiaowei Lu, Qian Zhang, Ruihuan Gu, Binghui Sun, Yijuan Sun

**Affiliations:** 1 Gynecology Department, The First Affiliated Hospital of Shandong First Medical University, NO.16766 Jingshi Road, Jinan, 250014 China; 2 Obstetrics Department, Shandong Provincial Third Hospital, No.12 Central Wuying Hill Road, Jinan, 250000 China; 3 Reproductive Medicine Centre, Obstetrics and Gynecology, Xinhua Hospital Affiliated to Shanghai Jiaotong University School of Medicine, Shanghai, 200092 China; 4 Shanghai Ji Ai Genetics & IVF Institute, Obstetrics and Gynecology Hospital, Fudan University, NO.588 Fangxie Road, Shanghai, 200011 China

**Keywords:** Hypoxia-inducible factor-2α, Endometrial receptivity, Adenomyosis, PT2399

## Abstract

**Background:**

Adenomyosis (AM) is an important cause of female infertility. However, the underlying mechanism remains unclear. This report describes a preliminary study of hypoxia and its possible association with endometrial receptivity in AM.

**Methods:**

The study was divided into in vitro and in vivo experiments. In vitro, expression levels of the endometrial receptivity markers HOXA10 and HOXA11 in the implantation period were examined using real-time PCR and western blotting. Endometrial expression of hypoxia-inducible factor (HIF)-1α, HIF-2α, and HIF-3α was determined using immunohistochemistry. In vivo, using an AM mouse model established by oral administration of tamoxifen, we inhibited expression of HIF-2α using an HIF-2α antagonist (PT2399; 30 mg/kg body weight, twice daily by oral gavage for 2 days) and then examined expression levels of Hoxa10 and Hoxa11 using real-time PCR and western blotting.

**Results:**

Endometrial mRNA and protein expression levels of HOXA10 and HOXA11 were significantly lower in patients with AM than in control patients. Expression of HIF-2α was significantly higher in the AM group than in the control group, whereas that of HIF-1α and HIF-3α was equivalent in both groups. In vivo analysis showed that administration of the HIF-2α antagonist resulted in increased expression of Hoxa10 and Hoxa11 at both the mRNA and protein levels in AM model mice.

**Conclusions:**

HIF-2α overexpression may be one reason for decreased endometrial receptivity in AM. The current findings provide insight into HIF-2α-mediated AM-related infertility and suggest that PT2399 has potential as a treatment for AM.

**Trial registration:**

This trial was retrospectively registered.

## Background

Adenomyosis (AM) is one of the most common disorders in women of reproductive age, with an incidence of 5–70% [1]. With improvements in diagnostic techniques, there is increasing evidence recently that AM is an important cause of female infertility [2]. One report indicated that 27% of infertile patients who received assisted reproductive technology (ART) treatment exhibited AM [3]. Implantation (odds ratio (OR) 0.66, 95% confidence interval (CI) 0.49–0.88) and clinical pregnancy (OR 0.75, 95% CI 0.61–0.93) rates are significantly lower among AM patients than for non-AM patients receiving ART treatment [4]. These findings show that AM may be frequent in infertile patients and may decrease the pregnancy rate; accordingly, AM has received considerable clinical attention in reproductive research.

It has been shown that AM may influence normal uterine peristalsis and sperm transport, leading to infertility and abortion [5]. The effects of AM on endometrial receptivity have become a key focus of research in recent years. Endometrial receptivity is a vital process for establishing a normal pregnancy. Molecular bioindicators for evaluating endometrial receptivity include homeobox A10 (HOXA10), HOXA11, integrin b3, leukaemia inhibitory factor (LIF), and osteopontin. One study reported that expression of HOXA10 and interleukin 10 (IL-10) in the eutopic endometrium was significantly reduced in patients with AM [6]. Using real-time polymerase chain reaction (Real-time PCR) and immunohistochemical techniques, Yen et al. found that LIF and its receptor were significantly decreased during embryo implantation in patients with AM [7]. Additionally, our preliminary experiments showed that HOXA10, HOXA11, integrin b3, and LIF expression and pinocytosis in the endometrium were significantly decreased in a mouse model of AM during the implantation period [8].

Hypoxia-inducible factors (HIFs) are important transcription factors that control the cellular adaptive responses to hypoxia, a core feature of hypoxia regulation [9]. Each HIF comprises two subunits, a functional α subunit and a structural β subunit. The HIF-α family includes HIF-1α, HIF-2α, and HIF-3α, which differ structurally in their α-subunits. Current research mainly focuses on HIF-1α, whereas HIF-2α has been less studied. Although HIF-1α and HIF-2α have similar structures, they have quite different physiological functions. HIF-1α is generally activated under acute hypoxia conditions, decreasing significantly with prolonged hypoxia. Conversely, HIF-2α levels increase significantly with prolonged hypoxia [10]. Additionally, the distribution of HIF-1α and HIF-2α differs throughout the body. HIF-1α is widely distributed in the body and expressed in various organs and tissues, but HIF-2α is mainly expressed in the endometrium and kidney, with the former suggesting that it may play a role in embryo implantation. Nonetheless, few studies have focused on the role of hypoxia in the uterus during implantation, and there is limited research on the role of HIF-2α as a hypoxia sensor.

The current study of the effect of a low oxygen microenvironment on endometrial expression of HIF-α factors in AM provides a new perspective for AM-related infertility.

## Methods

### Study design

This study included two parts. In part I, the in vitro study, we examined expression levels of the endometrial receptivity markers HOXA10 and HOXA11 in the implantation period using real-time (RT)-PCR and western blotting. Additionally, we determined endometrial expression of HIF-1α, HIF-2α, and HIF-3α using immunohistochemistry. In part II, the in vivo study, we performed a preliminary investigation into the effect of HIF-2α on endometrial receptivity using an AM mouse model established by oral administration of tamoxifen.

### Patient cohort

All patients were admitted to the Department of Gynaecology and Obstetrics, The First Affiliated Hospital of Shandong First Medical University for hysterectomy between January 2018 and December 2018. According to pathological results, 30 enrolled patients were divided evenly into two groups: an AM group (*n* = 15) and a non-AM group (*n* = 15). We used widely accepted standards for the pathologic diagnosis of AM, as follows: benign invasion of the endometrium into the myometrium, producing a diffusely enlarged uterus that microscopically exhibited ectopic, non-neoplastic endometrial glands and stroma surrounded by hypertrophic and hyperplastic myometrium [11]. No patients had received endocrine therapy, including oral contraception, gonadotropin-releasing hormone agonists, or hormone replacement therapy, for at least 6 months before this study. Informed consent was obtained from all patients.

### Mouse model of adenomyosis

Newborn female mice (Institute of Cancer Research strain) were randomly assigned to two groups, with 12 and 6 mice in the AM and non-AM groups, respectively. All mice were housed in a temperature-controlled room (22 °C) with controlled lighting (12-h light/dark cycle) and were given free access to water and a standard diet. The AM mouse model was established using tamoxifen, as previously reported by our group [8, 12]. Prior studies have also demonstrated the effectiveness of this approach [13, 14]. In this study, haematoxylin and eosin (H&E) and smooth muscle actin staining were used to confirm successful establishment of the mouse model of AM. The AM mice were fed tamoxifen at 2.7 μmol/kg of body weight (Shanghai Fudan Forward Science & Technology Co., Ltd., Shanghai, China) from postnatal day 2 to day 5. Four weeks after birth, the AM mice were randomly allocated to two groups, with 6 mice/group. Mice were administered 30 mg/kg PT2399 (an HIF-2α antagonist) twice daily by oral gavage for 2 days [15].

Six weeks after birth, each female was mated with one male overnight and checked for vaginal plugs the following morning. The day on which a vaginal plug was found was designated gestation day 1.

### Sample collection

Blood samples were collected before surgery. Serum cancer antigen 125 (CA125) levels were measured using a chemiluminescent microparticle immunoassay (Beckman Coulter Inc., Brea, CA, USA). The inter- and intra-assay coefficients of variation were all < 10%.

Endometrial samples were taken from patients during hysterectomy in the luteal phase of the menstrual cycle. Mice were euthanized at 19:00–20:00 h on day 4 of gestation (time of implantation). Endometrial tissue samples from patients and mice were each divided into two sections: one was fixed in 4% formalin solution, embedded in a paraffin block and cut into 4-μm sections for H&E pathological analysis and immunohistochemical analysis; the other was immediately snap frozen and stored at − 80 °C for subsequent analyses.

### Immunohistochemistry

The paraffin-embedded sections were dewaxed and rehydrated according to standard protocols, incubated with 3% H_2_O_2_ to eliminate endogenous peroxidase activity, and then blocked with 10% goat serum. The slices were incubated with anti-HIF-1α (Abcam, Cambridge, UK), **−**HIF-2α (Abcam), **−**HIF-3α (Abcam), or α-SMA (Wuhan Goodbio Technology, Wuhan, China) antibodies overnight at 4 °C. Appropriate conjugated secondary antibodies (Proteintech Group, Inc., Rosemont, IL, USA) were incubated with the tissue samples for 1 h at room temperature. For negative control samples, the primary antibodies were omitted. Quantitative analysis of immunohistochemical staining was performed using ImageJ (National Institutes of Health, Bethesda, MD, USA). Images were assessed by two investigators who were blinded to each other.

### Real-time PCR

We extracted total RNA from endometrial tissue using TRIzol reagent (TaKaRa, Dalian, China) according to the manufacturer’s instructions. First-strand cDNA was synthesized using primers designed by Sangon Biotech Co. Ltd. (Shanghai, China). SYBR Green-based genomic quantitative real-time PCR was performed using a 7500 Fast Real-Time PCR System (Thermo Fisher Scientific, Waltham, MA, USA). The sequences of the forward (F) and reverse (R) primers were as follows: Hoxa10, F-5′-GCCCCTTCAGAAAACAGTAAAG-3′, R-5′-AGGTGGACGCTACGGCTGATCTCTA-3′; Hoxa11, F-5′-TCCAGCCTCCCTTCTTTTTTG-3′, R-5′-GTAGCAGTGGGCCAGATTGC-3′.

The 2^−ΔΔCt^ method was used to calculate relative gene expression levels, and β-actin (*ACTB*) was employed for normalization of the expression data. Analysis of each sample was performed in triplicate.

### Western blotting

Proteins were extracted from the endometrium, and whole-cell extracts were analysed by western blotting. Equal amounts of protein samples were loaded onto 10% SDS-PAGE gels and transferred onto polyvinylidene difluoride membranes (EMD Millipore, Bedford, MA, USA). After being blocked with 5% non-fat milk in Tris-buffered saline with 0.05% Tween-20 for 1 h, the membranes were incubated overnight at 4 °C with primary antibodies against the following: Hoxa10 or Hoxa11 (both from Abcam). Following incubation with corresponding secondary antibodies for 2 h at room temperature, the protein bands were visualized using an ECL kit (Thermo Fisher Scientific). Protein band intensity was determined using ImageJ and Adobe Photoshop 5.0 (Adobe Inc., San Jose, CA, USA).

### Statistical analysis

Statistical analysis was performed using SPSS software (17.0) (SPSS Inc., Chicago, IL, USA). The Mann–Whitney test was used to compare age, body mass index (BMI), and CA125 level between the AM and control groups. Expression of HOXA10 and HOXA11 was also analysed using the Mann–Whitney test. Categorical variables were examined by using the Chi-square test. The results are presented as the mean ± standard deviation. *P* < 0.05 was considered statistically significant.

## Results

### Clinical characteristics of patients

No significant differences were observed with respect to age or BMI between the two groups. Hypermenorrhoea and dysmenorrhoea were significantly higher (*P* = 0.0001 and *P* < 0.0001, respectively; Table 1) in the AM group than in the control group, and AM patients exhibited higher CA125 levels (*P* < 0.0001; Table 1).

**Table 1:** Baseline characteristics of the patients

	The control group, *N* = 15	The adenomyosis group, *N*=15	*p* value
Age (years)	41.54±3.12	42.17±2.59	0.0943^#^
BMI (kg/m^2^)	22.60±3.88	23.12±2.13	0.3154^#^
Hypermenorrhea	2/15 (0.13%)	13/15 (0.87%)	0.0001^*^
Dysmenorrhea	1/15 (0.07%)	15/15 (100%)	<0.0001^*^
CA125	19.59±5.31	50.64±10.17	<0.0001^#^

* Chi-square test; #Mann-Whitney test

### Endometrial mRNA and protein expression levels of HOXA10 and HOXA11 during the implantation window

**Fig. 1:**
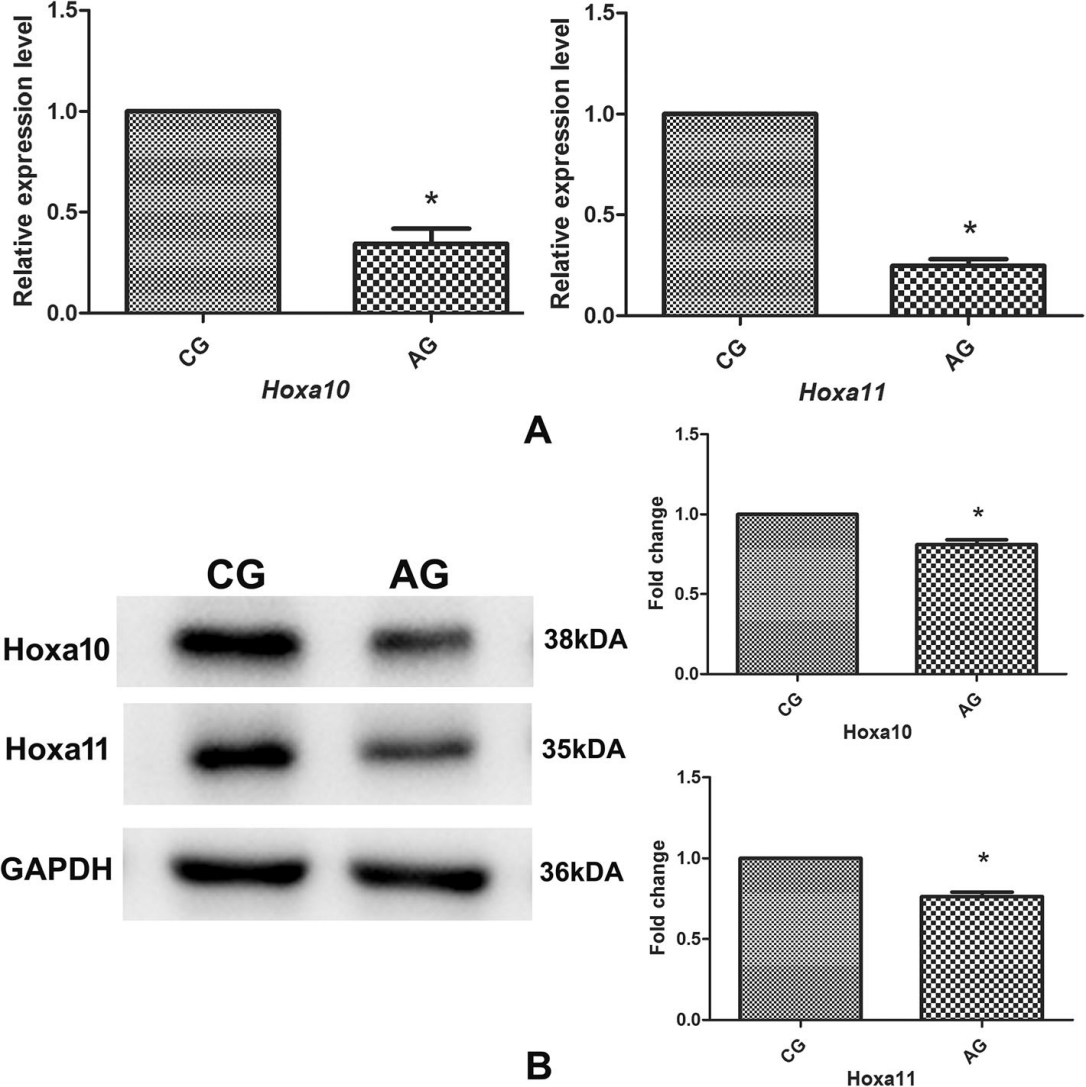
Hoxa10 and Hoxa11 mRNA and protein expression in the endometrium between the CG and the AG. **a** mRNA levels of Hoxa10 and Hoxa11. **b** Protein levels of Hoxa10 and Hoxa11. CG, control group; AG, adenomyosis group

mRNA expression levels of *HOXA10* and *HOXA11* were significantly lower in patients with AM than in control patients (*P* < 0.05; Fig. 1a). Western blotting revealed a protein expression pattern similar to the mRNA results (*P* < 0.05; Fig. 1b).

### Endometrial expression of the HIF-α family in the implantation period

To examine the relationship of the HIF-α family and endometrial receptivity in AM, we investigated expression of HIF-α members HIF-1α, HIF-2α, and HIF-3α during the implantation period using immunohistochemical staining and image analysis.

**Fig. 2:**
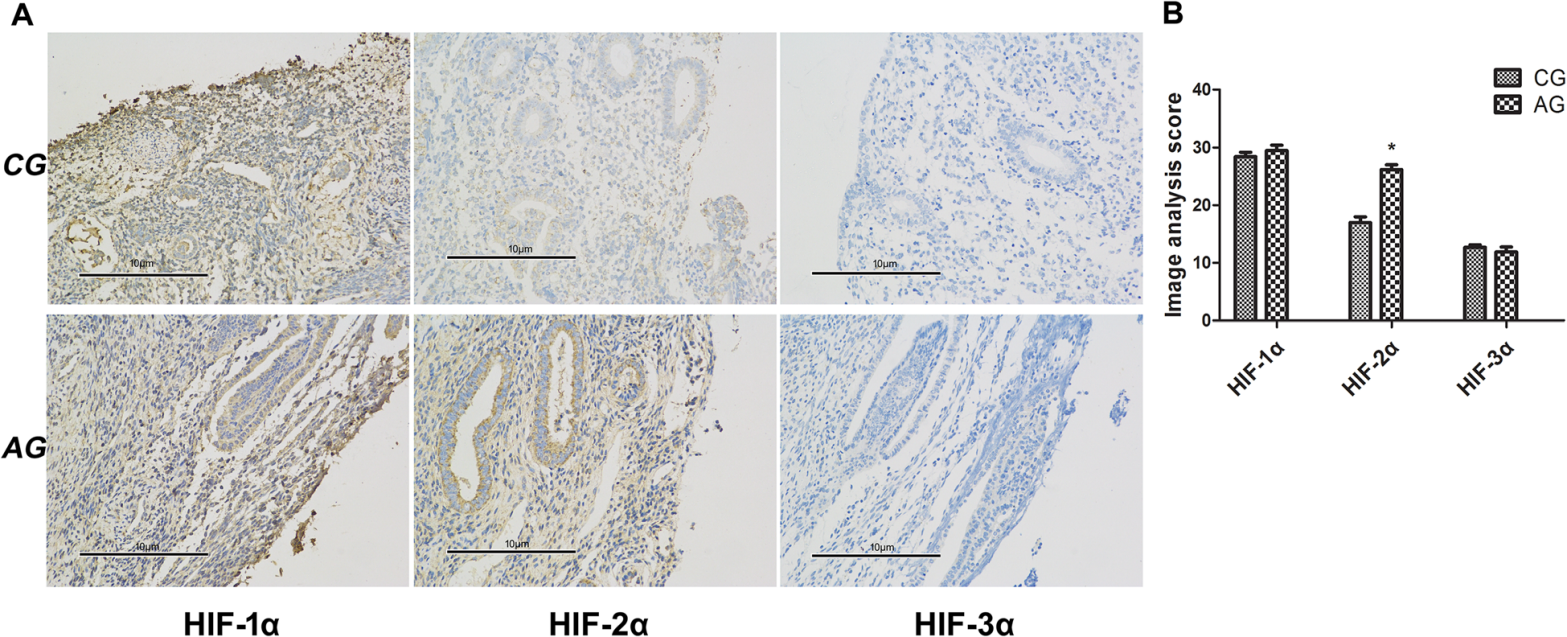
Immunohistochemistry of HIF-α members in the endometrium during the implantation window. **a** Representative images of HIF-1α, HIF-2α and HIF-3α (× 400 magnification). **b** Semi-quantitative analysis of HIF-1α, HIF-2α and HIF-3α protein expression. CG, control group; AG, adenomyosis group

We found that HIF-1α was highly expressed and that HIF-3α showed low expression in the luteal phase in both the AM and control groups, but with no significant differences between the two groups. HIF-2α expression increased significantly in the AM group compared with the control group (*P* < 0.05; Fig. 2), which suggests that HIF-2α may play an important role in embryo implantation in AM patients. This finding led to further study of its potential roles in endometrial receptivity.

### Effect of HIF-2α on endometrial receptivity in a mouse model of AM

**Fig. 3:**
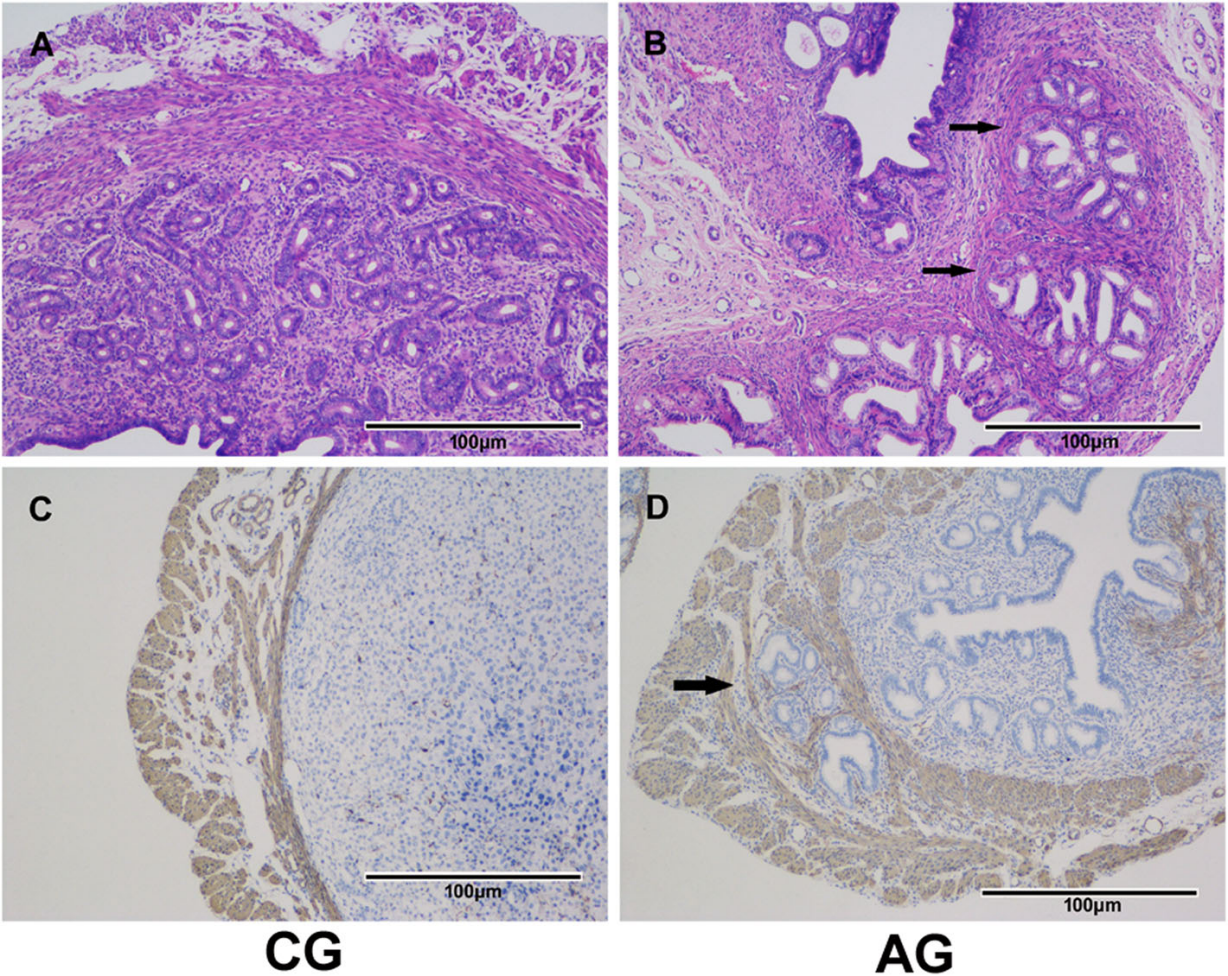
H&E and alpha-smooth muscle actin staining of mouse uterine tissue. Endometrial glands penetrated deeply into the myometrium beneath the endometrium of the uterus (arrow) in the AG (**b**, **d**). However, the endometrium was surrounded by regular, concentric layers of smooth muscle in the CG (**a**, **c**). CG, control group; AG, adenomyosis group

We used an established AM mouse model to evaluate the role of HIF-2α in endometrial receptivity in vivo. AM mice were established by treatment with tamoxifen between postnatal days 2 and 5. H&E and smooth muscle actin staining confirmed that the AM model was successfully established (Fig. 3).

**Fig. 4:**
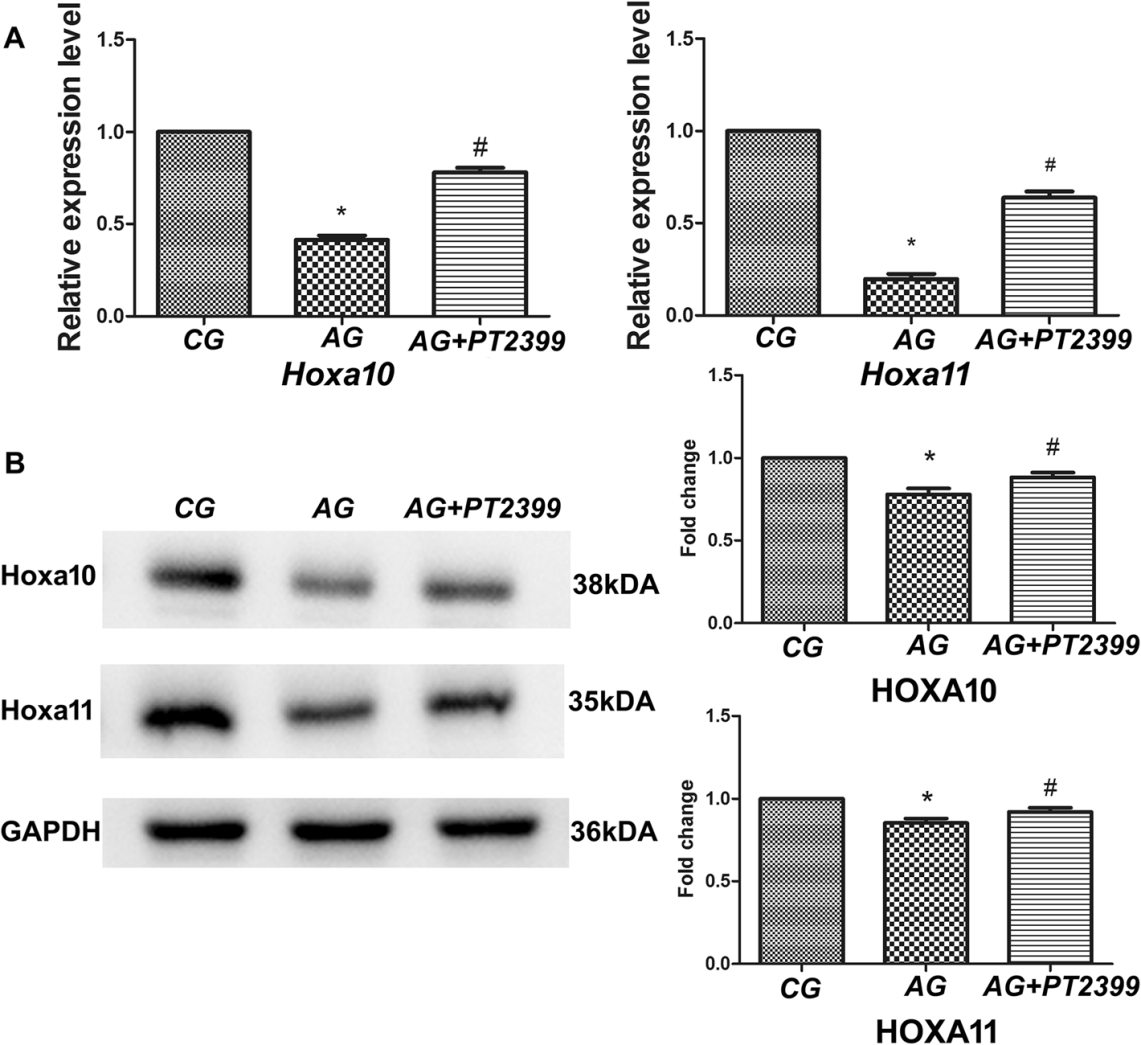
The impacts of HIF-2α on endometrial receptivity in AM. **a** mRNA levels of Hoxa10 and Hoxa11. **b** Protein levels of Hoxa10 and Hoxa11. CG, control group; AG, adenomyosis group; AG+PT2399, adenomyosis group with PT2399 administration

As illustrated in Fig. 4, the degree of positive staining for Hoxa10 and Hoxa11 was lower in AM tissue than in control tissue (*P* < 0.05). Moreover, administration of PT2399 to the AM model mice resulted in reduced HIF-2α expression and increased expression of Hoxa10 and Hoxa11 at both the mRNA and protein levels (*P* < 0.05). These results indicate that endometrial HIF-2α may affect embryo implantation in AM.

## Discussion

HOXA10 and HOXA11 are important molecular markers of endometrial receptivity and play an important role in the process of embryo implantation and endometrial decidualization [16]. In this study, we compared the endometrial receptivity of AM and control groups using RT-PCR and western blot analyses. The results showed that expression of HOXA10 and HOXA11 was significantly lower in patients with AM than in controls. These findings confirmed the results of our previous study, in which we found that expression of endometrial receptivity markers was significantly decreased during the embryo implantation period in an established animal AM model [8].

To date, the precise mechanism of embryo implantation remains obscure. The hypoxic microenvironment has been a focus of research in recent years. Some studies have shown that a hypoxic microenvironment is closely related to female fertility; for example, low O_2_ might regulate placental development [17], and hypoxia might induce oocyte autophagy [18]. Thus far, no studies have reported the relationship between a hypoxic microenvironment and endometrial receptivity in AM.

In this study, we selected the hypoxic microenvironment as the cut-in point. By analysing HIF-α family expression in the luteal phase, we discovered differential expression of HIF-2α between AM and non-AM groups. These findings suggest that HIF-2α affects embryo implantation in AM. To test this hypothesis, we investigated HIF-2α expression in vivo using artificial induction of AM in mice. Based on RT-PCR and western blot analyses, we observed increased endometrial expression of HIF-2α in AM mice; inhibition of HIF-2α significantly increased expression of endometrial receptivity markers. These findings indicate that HIF-2α may function to reduce endometrial receptivity.

The endometrium has complex requirements for oxygen that differ before and after embryo implantation. It has been reported that before embryo implantation, the concentration of dissolved oxygen is relatively low in the endometrium surface compared with the inner portion of the endometrium and the myometrium. The endometrium is further away from the uterine blood vessels, and oxygen concentration diminishes with distance from blood vessels [19]. Moreover, embryonic development occurs within a hypoxic environment before implantation [20]. The hypoxic environment of the preimplantation endometrium is suitable for human embryogenesis, though the oxygen concentration rises markedly in the endometrium after embryo implantation and angiogenesis [21], resulting in an aerobic environment that provides sufficient oxygen for embryonic development. Therefore, the initial hypoxic microenvironment of the endometrium changes over time, resulting in altered physiological function. We propose that in AM, oxygen levels in the endometrium remain very low, even after embryo implantation. This hypoxic microenvironment cannot provide enough oxygen for normal embryo development and eventually leads to implantation failure.

The HIF-α family includes key regulators of the transcriptional response to oxygen deprivation, and much attention has been paid to their physiological and pathological functions. Although HIF-1α and HIF-2α belong to this family, they have distinct expression patterns and biological functions [22]. In this study, we found that HIF-1α was highly expressed and that HIF-3α was lowly expressed in the luteal phase of both the AM and control groups. We also detected differential expression of HIF-2α between the two groups. There have been similar studies of other diseases, but the results have not been consistent. For example, Zhang et al. found that HIF-1α expression was significantly higher in the eutopic endometrium of endometriosis than in the normal endometrium [23]. Another study found that HIF-1α improved endometrial receptivity in patients with polycystic ovary syndrome [20]. Considering the above research, we believe that distinct HIF-α factors may have different functions in different diseases, highlighting the need for further systematic research and analysis.

Previous studies have demonstrated a close relationship between HIF-2α and angiogenesis. Feng et al. reported that HIF-1α and HIF-2α were upregulated in gastrointestinal vascular malformations, which can induce vessel formation [24]. Additionally, HIF-2α correlated positively with vascular endothelial growth factor (VEGF) at both the mRNA and protein levels in primary osteoarthritic cartilage [25]. According to the study of Zhang et al., expression of HIF-2α and VEGF mRNA was significantly elevated in cervical squamous cell carcinoma compared with controls, with a significant positive correlation between them [26]. It has also been reported that VEGF is overexpressed in the eutopic endometrium in AM, which may lead to menorrhagia [27, 28]. In this study, we found that expression of HIF-2α was markedly higher in the AM group than in the control group. Considering our findings and the literature reports, we propose that abnormally high HIF-2α expression may induce VEGF production and lead to heavy menstrual bleeding. Whether HIF-2α regulates endometrial receptivity by modulating angiogenesis needs further study. If this hypothesis is confirmed, the HIF-2α antagonist PT2399 might be used to treat infertility in patients with AM and reduce menstrual blood loss.

Embryo implantation and early embryo development are complicated. The precise role of HIF-2α factors in this process remains unclear. One study showed implantation failure in mice after disruption of HIF-2α in the whole uterus. Supplementation with progesterone and LIF was able to restore decidualization and normal embryo implantation location but failed to reduce pregnancy failure [21]. Such findings further indicate that more than one mechanism may be involved in this process, such as luteal phase deficiency and abnormal inflammation.

Our study is not without limitations. One potential weakness is the small sample size, which may have induced selection bias and confounding bias in the statistical analysis. Additionally, potential reasons for elevated HIF-2α levels in AM patients (e.g., immunological, genetic, and hormonal) were not evaluated. Finally, outcomes related to endometrial receptivity were not examined functionally in the current study, and further investigations are required to confirm this issue (e.g., by assessing implantation rate and pregnancy rate).

## Conclusions

In this study, we showed that abnormal expression of HIF-2α may be one reason for decreased endometrial receptivity in patients with AM. The findings provide new insight into HIF-2α-mediated AM-related infertility and the possible application of the HIF-2α antagonist PT2399 as treatment. To explore the underlying mechanisms, future studies should attempt to identify potential target genes and biological pathways of HIF-2α actions. For example, HIF-2α may play a role in embryo implantation by activating the Wnt and Notch pathways. Beyond that, it will be important to study the effects of a hypoxic microenvironment on stromal and glandular cells of the human endometrium.

## Data Availability

The datasets used and analysed during the current study are available from the corresponding author on reasonable request.

## Abbreviations

AM: Adenomyosis
HIF-α: Hypoxia inducible factor-alpha;
ART: Assisted reproductive technology
CI: Confidence interval
HOXA10: Homeobox A10
LIF: Leukaemia inhibitory factor
IL-10: Interleukin 10
HIFs: Hypoxia-inducible factors
GnRH: Gonadotropin-releasing hormone
BMI: Body mass index
VEGF: Vascular endothelial growth factor
H&E: Haematoxylin and eosin; *ACTB:* β-Actin
